# DELMEP: a deep learning algorithm for automated annotation of motor evoked potential latencies

**DOI:** 10.1038/s41598-023-34801-9

**Published:** 2023-05-22

**Authors:** Diego Milardovich, Victor H. Souza, Ivan Zubarev, Sergei Tugin, Jaakko O. Nieminen, Claudia Bigoni, Friedhelm C. Hummel, Juuso T. Korhonen, Dogu B. Aydogan, Pantelis Lioumis, Nima Taherinejad, Tibor Grasser, Risto J. Ilmoniemi

**Affiliations:** 1 Institute for Microelectronics, Technische Universität Wien, Gußhausstraße 27-29/E360, 1040 Vienna, Austria; 2 Department of Neuroscience and Biomedical Engineering, Aalto University School of Science, Espoo, Finland; 3 BioMag Laboratory, HUS Medical Imaging Center, University of Helsinki, Aalto University and Helsinki University Hospital, Helsinki, Finland; 4 School of Physiotherapy, Federal University of Juiz de Fora, Juiz de Fora, MG Brazil; 5 Department of Neurology, Stanford University School of Medicine, Stanford, CA USA; 6 Department of Psychiatry and Behavioral Sciences, Stanford University School of Medicine, Stanford, CA USA; 7 Defitech Chair of Clinical Neuroengineering, Neuro-X Institute (INX) and Brain Mind Institute (BMI), École Polytechnique Fédérale de Lausanne (EPFL), 1202 Geneva, Switzerland; 8 Defitech Chair of Clinical Neuroengineering, Neuro-X Institute (INX) and Brain Mind Institute (BMI), Ecole Polytechnique Fédérale de Lausanne (EPFL Valais), Clinique Romande de Réadaptation, 1951 Sion, Switzerland; 9 Clinical Neuroscience, Geneva University Hospital (HUG), 1205 Geneva, Switzerland; 10 A. I. Virtanen Institute for Molecular Sciences, University of Eastern Finland, Kuopio, Finland; 11 Institute for Computer Technology, Technische Universität Wien, Vienna, Austria; 12 Institute of Computer Engineering, Heidelberg University, Heidelberg, Germany

**Keywords:** Neuroscience, Data processing

## Abstract

The analysis of motor evoked potentials (MEPs) generated by transcranial magnetic stimulation (TMS) is crucial in research and clinical medical practice. MEPs are characterized by their latency and the treatment of a single patient may require the characterization of thousands of MEPs. Given the difficulty of developing reliable and accurate algorithms, currently the assessment of MEPs is performed with visual inspection and manual annotation by a medical expert; making it a time-consuming, inaccurate, and error-prone process. In this study, we developed DELMEP, a deep learning-based algorithm to automate the estimation of MEP latency. Our algorithm resulted in a mean absolute error of about 0.5 ms and an accuracy that was practically independent of the MEP amplitude. The low computational cost of the DELMEP algorithm allows employing it in on-the-fly characterization of MEPs for brain-state-dependent and closed-loop brain stimulation protocols. Moreover, its learning ability makes it a particularly promising option for artificial-intelligence-based personalized clinical applications.

## Introduction

**Figure 1:**
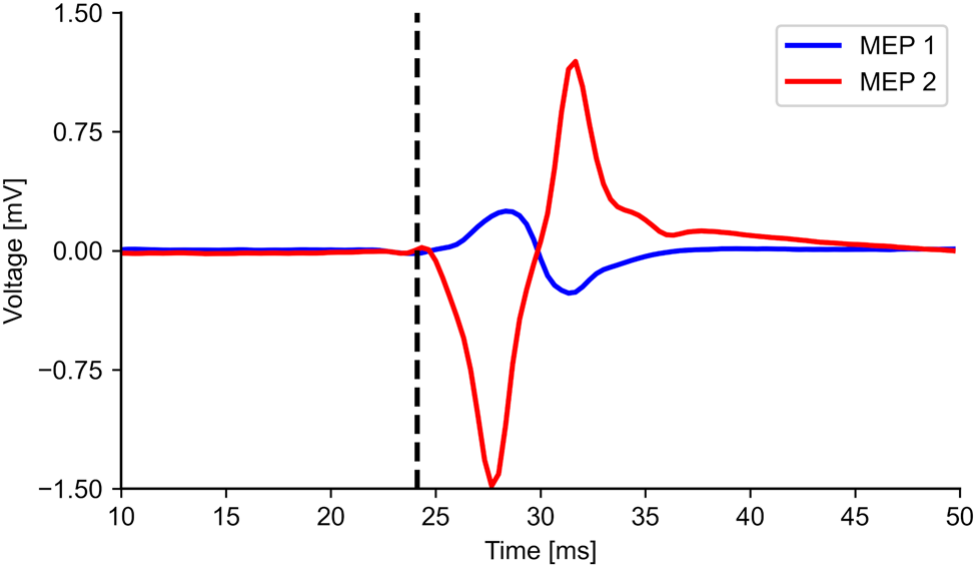
Two different MEP waveforms (red and blue curves) with similar latencies (black vertical line), defined as the time elapsed between the TMS and the beginning of the MEP trace. The epoch starts at the time the TMS pulse is delivered.

The motor evoked potential (MEP) generated by transcranial magnetic stimulation (TMS) is a crucial neurophysiological signal in research and clinical practice. MEP amplitude and latency allow us to assess quantitatively the corticospinal excitability. This is necessary to evaluate patients undergoing surgery and to monitor neuromotor diseases, such as the progression of multiple sclerosis^1^, the recovery of stroke patients^2^ and idiopathic generalized epilepsy patients^3^, among a wide range of medical applications. MEPs are commonly characterized by their latency, which is defined as the time elapsed between the stimulation and the onset of the MEP (Fig. 1). The MEP latency is usually annotated manually after visual inspection of the electromyography (EMG) recording, making the process time-consuming, operator-dependent, and prone to errors^4,5^. An algorithm to automate the characterization of MEPs would not only save time and reduce human errors, but would also boost the development of brain-state-dependent and closed-loop brain stimulation protocols by allowing accurate real-time MEP assessment^6^.

Several attempts have been made to develop algorithms to automate the MEP latency annotation. These algorithms are based either on absolute hard threshold estimation (AHTE)^7^ or on statistical measures^8,9^. A review and comparison of previous methods is presented by Šoda et al.^10^, together with their own algorithm named Squared Hard Threshold Estimator (SHTE). In general, the previously presented algorithms require the user to specify a set of so-called *magic numbers*. These are hyperparameters with a large impact on the algorithm performance, which are empirically derived and depend on the user's knowledge and experience^10^. On the other hand, Bigoni’s method^11^, which is a derivative-based algorithm, does not require the user to specify magic numbers.

Developing an algorithm to automate the annotation of MEPs is not trivial. Even in ideal conditions of high signal-to-noise ratio (SNR), MEPs are highly variable, presenting significant inter- and within-subject amplitude variability^12–14^. Similarly, the MEP latency variability is well known and has been previously documented for neurosurgical patients^12,15^. Under similar circumstances, the amplitude of two MEPs can differ up to an order of magnitude and present totally different shapes, as shown in Fig. 1. Furthermore, low-amplitude MEPs, commonly recorded in inhibitory stimulation paradigms with paired-pulse TMS have inherently lower SNR than high-amplitude MEPs, thus adding a new layer of complexity. Overall, these factors make it demanding to assess the MEP latency automatically and reliably.

In this context, machine-learning-based algorithms, particularly those employing deep learning techniques, offer a promising approach to provide an accurate and reliable solution. The MEP latency annotation is a pattern recognition problem, where deep learning methods have already demonstrated their potential^16^. In this work, we present our DELMEP algorithm, which relies on deep learning for automated MEP latency annotation. We contend that DELMEP will speed up data analysis procedures and facilitate the development of closed-loop brain stimulation protocols, as well as the development of personalized medical solutions. To the best of our knowledge, this is the first deep learning solution to the problem of automating the MEP latency estimation.

## Material and methods

### MEP dataset

We utilized a dataset collected from 9 healthy volunteers (3 women and 6 men, mean age: 30 years, range 24–41) for two studies^17,18^, which describe the detailed experimental protocol and stimulation paradigms. Experiments were performed in accordance with the Declaration of Helsinki and approved by the Coordinating Ethics Committee of the Hospital District of Helsinki and Uusimaa. All participants gave written informed consent before their participation.

TMS was applied with a monophasic trapezoidal waveform by our custom-made multi-channel TMS (mTMS) power electronics^19^ connected to a 2-coil transducer capable of electronically rotating the peak induced electric field^17^. EMG signals were digitized using an eXimia EMG 3.2 system (Nexstim Plc, Finland; sampling frequency 3 kHz; 10–500 Hz band-pass filter). MEPs were collected with single-pulse and paired-pulse paradigms. The paired-pulse stimuli were delivered with interstimulus intervals of 0.5 and 1.5 ms (short-interval intracortical inhibition, low-amplitude MEPs) and 6.0 and 8.0 ms (intracortical facilitation, high-amplitude MEPs). The conditioning stimulus intensity was 80% of resting motor threshold and the test stimulus and single pulse intensity were both 110% of resting motor threshold. MEPs were recorded from the abductor pollicis brevis, abductor digiti minimi and first dorsal interosseous muscles. EMG recordings showing muscle pre-activation or movement artifacts greater than ± 15 µV within 1 s before the TMS pulse were removed from the analysis. The raw MEPs were visually inspected, and the latency was manually annotated by a single expert (doctoral candidate; 7 years of experience) and quality-checked by a second expert (postdoctoral researcher; 10 years of experience) who confirmed the latency annotations. We note that the aforementioned experts are co-authors of this study. However, the dataset was collected and the latencies were annotated for two prior studies^17,18^ conducted before the conceptualization and development of DELMEP. Therefore, the annotations were performed independently of the development of the DELMEP algorithm. We performed an additional validation on an external MEP dataset annotated by three experts. From the three experts, one is a co-author of the present study. Data preprocessing and annotation was performed with custom-made scripts written in MATLAB R2017a (MathWorks Inc, USA). A total of 33,060 MEPs were recorded, i.e., 11,020 from each muscle group (abductor pollicis brevis, abductor digiti minimi, and first dorsal interosseous). From all MEPs, 232 (0.7%) were discarded because of pre-activation and 11,548 (34.9%) were discarded because of noise or no-response. Out of the remaining 21,244 MEPs, the validator discarded 4569 (21.5%) and approved 16,675 (78.5%). Therefore, in total, the dataset is composed of 16,675 MEPs and their peak-to-peak amplitudes and latencies.

### Latency estimation algorithm

**Figure 2:**
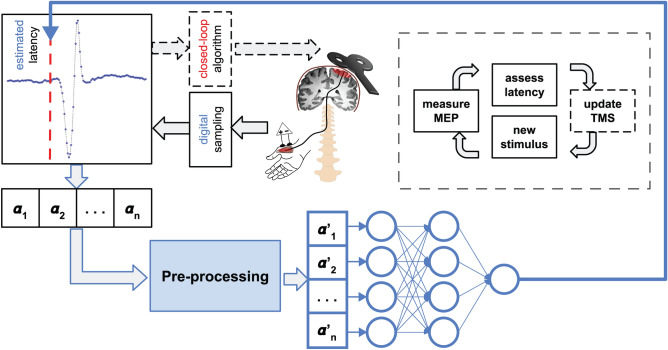
Workflow for automated assessment of MEP latencies in a possible closed-loop TMS set-up. MEPs are measured with electrodes placed on the target muscle and stored in a 120-dimension vector. The pre-processing is done by trimming, smoothing, centering, and normalizing the MEP. The resulting vector is used as an input to the neural network for the latency estimation. The dashed arrows show how our DELMEP algorithm could be applied to a closed-loop protocol (dashed box), in which the brain stimulation parameters are modified depending on the MEP responses.

To automate the MEP latency assessment, we developed an algorithm named DELMEP, written in Python 3.8 and available at https://github.com/connect2brain/delmep. The DELMEP pipeline is composed of the following steps (Fig. 2): (1) pre-processing and (2) latency estimation with a neural network. We present the details of each step below.

Pre-processing (step 1): The pre-processing simplifies the training and use of our neural network. Without the pre-processing, the high variability and noise of the MEPs would require that the neural network “learns” different inputs (MEP traces) corresponding to similar outputs (latencies). Here, the MEPs are represented by a mathematical vector of the raw voltage measurements, significantly reducing the complexity and increasing the speed of deep learning algorithms necessary to process the data. Hence, in this step, the data are (1) trimmed, (2) smoothed, (3) centered, and (4) amplitude normalized. The MEPs are trimmed from 10 to 50 ms after the TMS (120 samples). This is done to reduce and standardize their length, because in the resting condition, the measurements shortly after the TMS and much later than the end of the MEP do not carry relevant information. On the contrary, their inherent noise could pose a problem to the training and use of the neural network, since it would unnecessarily increase the dimensions of the input vectors.

**Figure 3:**
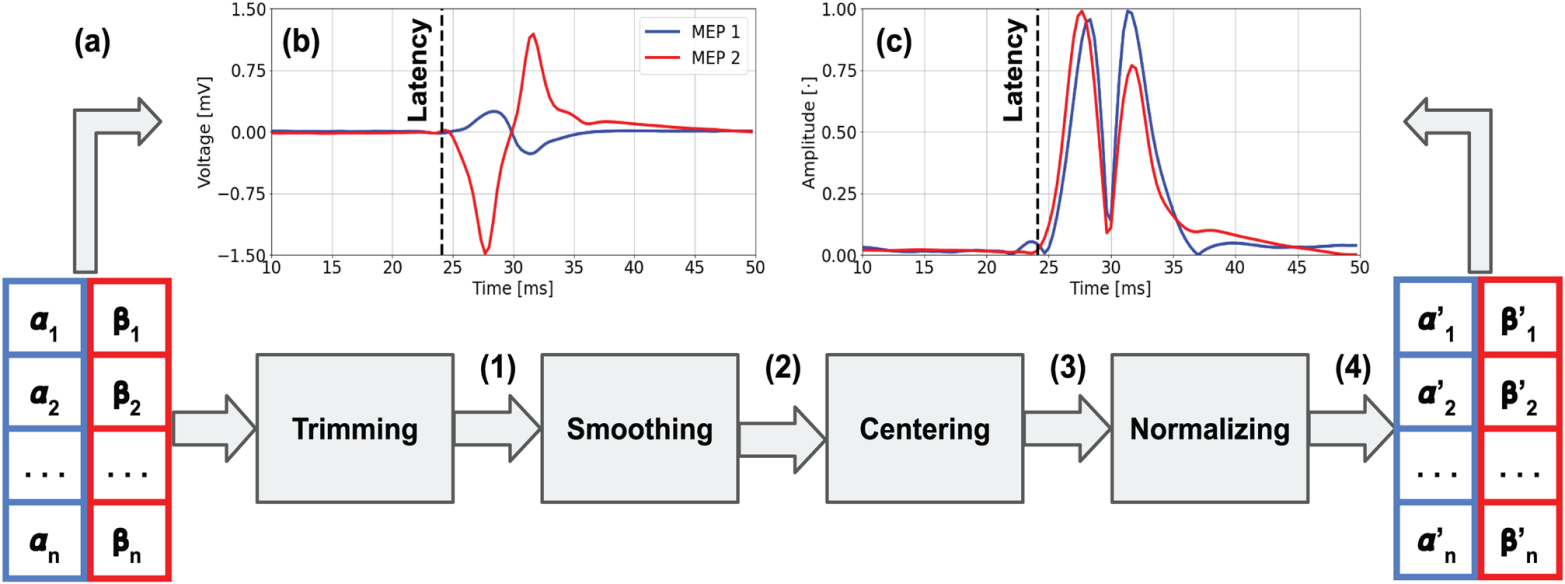
(**a**) Pre-processing of the two MEPs shown in Fig. 1, divided into: trimming (1), smoothing (2), centering (3) and normalizing (4). Panels (**b**) and (**c**) show the MEPs before and after the pre-processing, respectively. When comparing (**b**) and (**c**), note that the different MEPs look similar after the pre-processing, thus facilitating the training and later use of the neural network.

After trimming, the MEPs are smoothed with a moving average filter with a window length of 3 samples, to reduce the high-frequency noise of the recordings^20^. Next, the MEPs are centered by computing their mean value in the first 15 samples (5 ms) and then subtracting it from every sample. This step reduces the impact of low-frequency noise in the measurements, by counteracting the shifting it produces in the mean MEP value. The window length in the smoothing step and the time window in the centering step were tuned employing a grid search algorithm. Lastly, we normalize the MEPs so that their minimum and maximum values correspond to 0 and 1, respectively, to mitigate the effects of the large variations in amplitude. A detailed representation of this preprocessing is illustrated in Fig. 3, where the changes on the two MEPs presented in Fig. 1 are shown.

Deep learning algorithm (step 2): The pre-processed MEPs are used as inputs to the neural network, which produces a latency prediction as its output. This neural network is built as a multi-layer fully connected perceptron layout with two hidden layers of 30 artificial neurons each, and an output layer. We used the rectified linear unit activation function and trained the network with the Adam optimizer^21^ (early stopping criteria: 200 epochs; batch size: 32), as implemented in the software package Keras 2.4.3^22^.

### Data analysis and method validation

From all MEPs, 2113 (13%) are low amplitude (peak-to-peak amplitude (*V*_PP_) ≤ 100 µV), 2995 (18%) are medium amplitude (100 µV < *V*_PP_ ≤ 200 µV) and 11,565 (69%) are high amplitude (*V*_PP_ > 200 µV). The MEPs were first divided randomly into a training (13,340 MEPs) and testing (3335 MEPs) dataset, with a training/testing ratio of 80/20. We verified the accuracy and repeatability of our method by using it to evaluate the latency of the 13,340 and 3335 MEPs in the training and testing datasets, respectively, and comparing these results with the manual assessment of the expert. The comparison was made by computing the mean absolute error (MAE) between the latencies provided by our method and those provided by the expert. We analyzed the latency prediction error by computing the correlation of the automated latency estimate with the two main MEP features: *V*_PP_ and the manually annotated latency. We also estimated the computational times for the DELMEP algorithm using a standard desktop computer (CPU Intel Core i7-5650U 2.2 GHz and 8 GB of RAM).

For comparison, we used the following algorithms: Signal Hunter^9^, AHTE^10^, SHTE^10^ and Bigoni’s method^11^ to estimate the latency of the MEPs in the testing dataset. These estimations were then compared to the manual annotations of the expert and the MAE was computed for every method. Signal Hunter is an open source software for MEP analysis having a latency estimation algorithm based on statistical measures, it performs a moving average filtering on the MEP, differentiates the smoothed signal, calculates the standard deviation (SD), and finds the index value for which the difference between the absolute differentiated MEP value and the SD is the largest; thereafter, it estimates the MEP latency by subtracting a user-selected *magic number* from that index value. We implemented Signal Hunter with a magic number equal to 5, following the author’s original implementation^9^. The AHTE algorithm performs an absolute value operation on the MEP, finds its maximum amplitude and determines the threshold value (*V*_thr_), marks ± 10% around the mean value of the MEP, and finds the index value where the marked line is crossed by the MEP for the first time. The latency estimation is obtained by subtracting a user-selected magic number from that index value. The SHTE algorithm is based on the same principle as the AHTE algorithm, but it works by squaring the MEP coefficients, instead of performing an absolute value operation. We implemented the AHTE and SHTE algorithms (*V*_thr_ = 10% and magic number = 5) as done in^10^. Bigoni’s method is a derivative-based method, it reduces the MEP to a window of 10–50 ms after the stimulation, finds the peak and trough of the MEP, performs an absolute value operation, computes the approximate first derivative of the MEP until the peak, finds the longest vector of consecutive samples having a positive derivative, and estimates the latency as the first sample of this vector. All algorithms were implemented in Python 3.8.

To evaluate the generalizability of our DELMEP algorithm, we performed a cross-validation both within and across subjects. In a within-subject test, the data of each subject was split in 5 folds; 80% of the MEPs were used as training dataset and the remaining 20% as the validation set, interchangeably. Final results were obtained by computing the average MAE and SD of MAE across folds for each subject separately. The inter-subject variability of our DELMEP algorithm was tested using a leave-one-subject-out approach. In this test, the data from all but one subject was used to train the model and the MAE was estimated using the data from the left-out subject. This was repeated for all subjects and the MAE was computed in every iteration.

An additional validation was performed in which our DELMEP algorithm and Bigoni’s method were used to estimate the latency of the MEPs in an independent dataset, which is composed of 1561 MEPs and described in detail in the study by Bigoni et al.^11^. This dataset was collected from 16 healthy volunteers (eight women and eight men; age: 26.7 ± 2.6 years). The latencies were manually annotated by three different experts (with 0.5, 5 and 14 years of experience) who did not take part in the development of DELMEP. For validating DELMEP, we computed the ground truth (GT) latency as the mean value from the three annotations. About 99% of the MEPs in this dataset have a high amplitude (*V*_PP_ > 100 µV), as these MEPs were collected using a single-pulse paradigm, with a test-intensity chosen to produce MEP amplitude of 0.50 mV.

## Results

**Figure 4:**
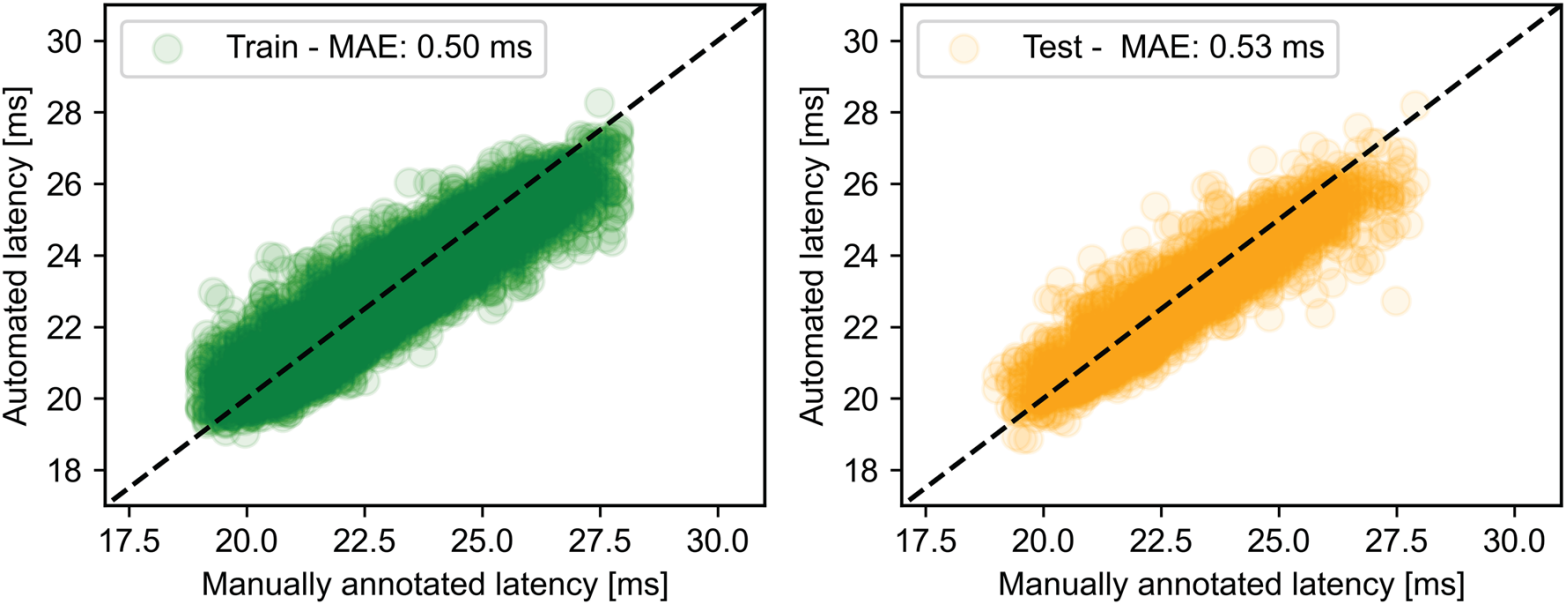
Automated MEP latency annotations with the proposed DELMEP algorithm in the training dataset (green) and testing dataset (orange). The results are compared to the manually assessed values. The MAE is presented for both datasets.

Training the neural network on a dataset of 13,340 MEPs required about 2 min and pre-processing an MEP trace required 1.2 ms. On average, annotating a pre-processed MEP required 65 µs. The estimated MEP latencies by the DELMEP algorithm and corresponding MAE, for the testing and training datasets are illustrated in Fig. 4. The similarity between the automated DELMEP and the manual expert annotation suggests a successful training process, since the MEPs in the testing dataset were not used to train the neural network.

**Figure 5:**
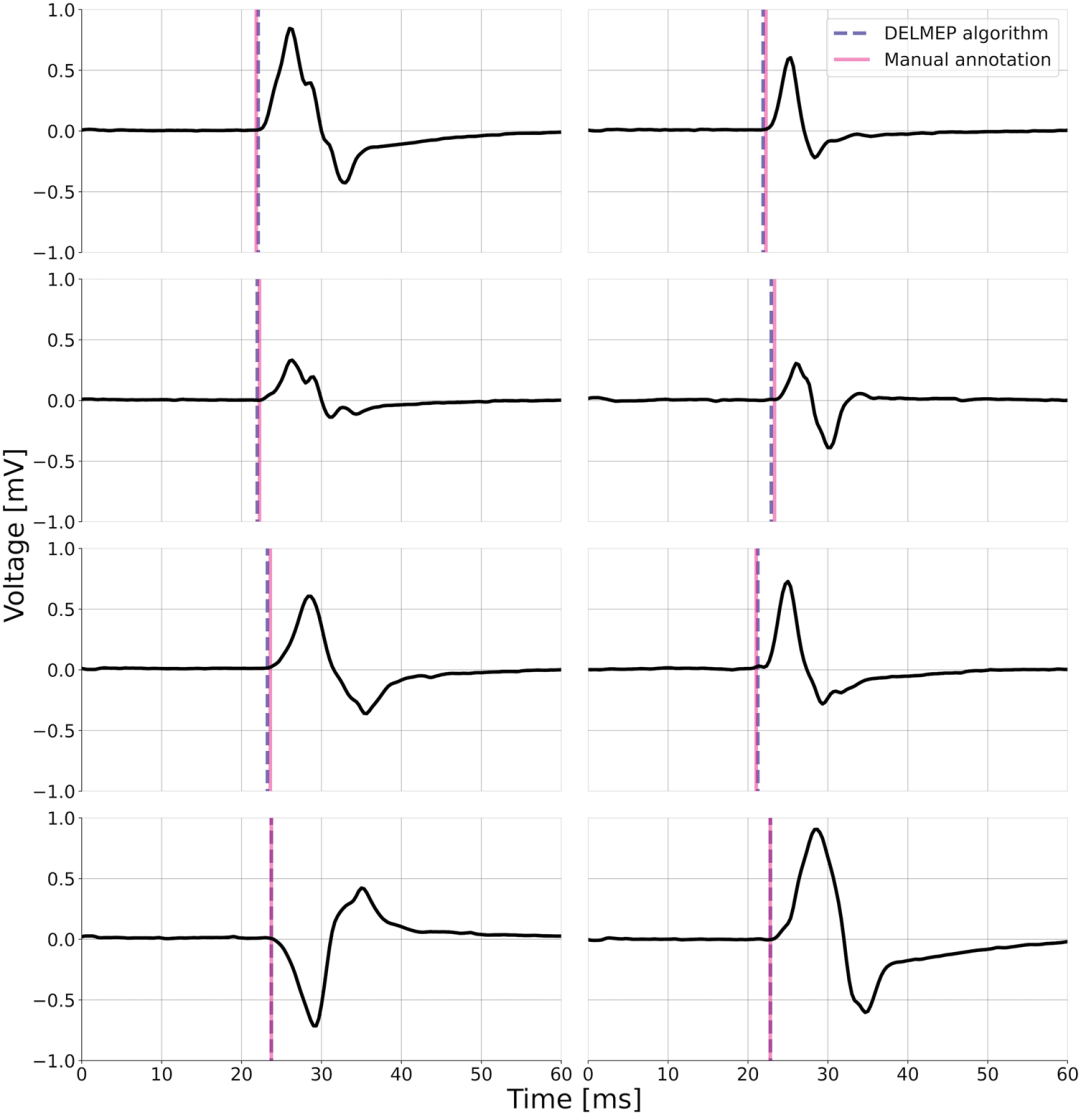
Illustrative MEPs from the testing dataset and their corresponding automated (dashed violet vertical line) and manually assessed (purple vertical line) latencies. These MEPs were not used to train the neural network. The similarity between both latencies indicates the successful performance of our DELMEP algorithm.

To provide a practical example of the DELMEP performance, Fig. 5 shows eight MEPs and their corresponding automated and manually annotated latencies. Although such a small sample can only be considered as an illustrative example, it provides a notion of how the proposed algorithm performs when used to replace a human expert in MEP annotations.

**Figure 6:**
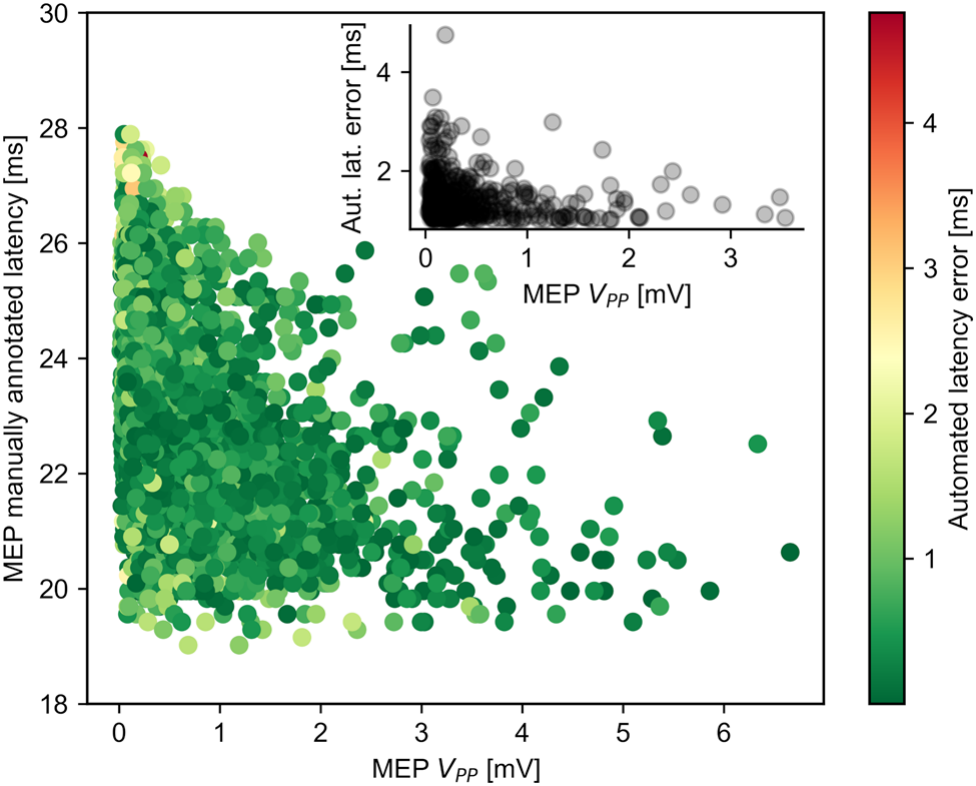
Map of the DELMEP algorithm errors in estimating MEP latencies, as a function of the MEP *V*_PP_ amplitude and manually annotated latency. The upper-right panel shows the DELMEP estimation error versus the MEP *V*_PP_ amplitude, for MEPs with latency estimation error higher than 1 ms.

Figure 6 illustrates the error associated with the DELMEP algorithm for the corresponding MEP *V*_PP_ and manually annotated latency. The sub-panel shows the correlation between the DELMEP latency estimation error and the MEP *V*_PP_, for MEPs with an estimation error equal or higher than 1 ms. From the 3,335 MEPs assessed by DELMEP in the testing dataset, 1,895 (57%) had an error lower than 0.5 ms and 2924 (88%) had an error lower than 1 ms, making it useful not just in research but also in clinical medical practice.

The results from the validation comparison between DELMEP and Signal Hunter, AHTE, SHTE, and Bigoni’s method can be found in Table 1, where the MAE is reported for the entire testing dataset and also divided between high- and low-amplitude MEPs. It is important to notice that Bigoni’s method discards MEPs when it is not able to find a long enough vector of samples with positive derivatives. The minimum number of samples with positive derivatives in our implementation was set to five, following the original author’s implementation^11^. This resulted in 483 out of 3335 MEPs in the testing dataset (15%), most of which corresponded to low-amplitude MEPs, being discarded by Bigoni’s method. To make a direct comparison, only the remaining MEPs were considered to compute the MAE of every method. However, we note that for the cross-validation, in Table 2, the entire dataset was used.

**Table 1:** Comparison of mean absolute errors (MAE) obtained with DELMEP, Signal Hunter, AHTE, SHTE, and Bigoni’s method for the MEPs in the testing dataset. The MAE is reported for the entire dataset and for low/high amplitude MEPs. MEPs discarded by Bigoni’s method were also discarded on the other methods for a direct comparison.

Method	MAE—low amplitude MEPs (*V*_PP_ ≤ 100 µV) [ms]	MAE—high amplitude MEPs (*V*_PP_ > 100 µV) [ms]	MAE—all MEPs [ms]
Signal hunter	22.9	6.3	7.7
AHTE	16.5	2.8	4.0
SHTE	7.3	1.3	1.8
Bigoni’s method	1.0	0.8	0.8
DELMEP	0.6	0.5	0.5

**Table 2:** Cross-validation of DELMEP across the entire dataset. The available data was divided into five different batches. In each test, one of the batches was used to test the algorithm and the remaining batches to train it. The mean absolute error (MAE) is reported for every batch, as well as its average and standard deviation (SD) for all batches.

Cross-validation MAE [ms]					Average MAE [ms]	SD [ms]
1	2	3	4	5		
0.5	0.5	0.5	0.5	0.6	0.5	0.03

The resulting MAE from the five-fold cross-validation when using each batch for testing is reported in Table 2, together with the average MAE for all tests and its SD.

**Figure 7:**
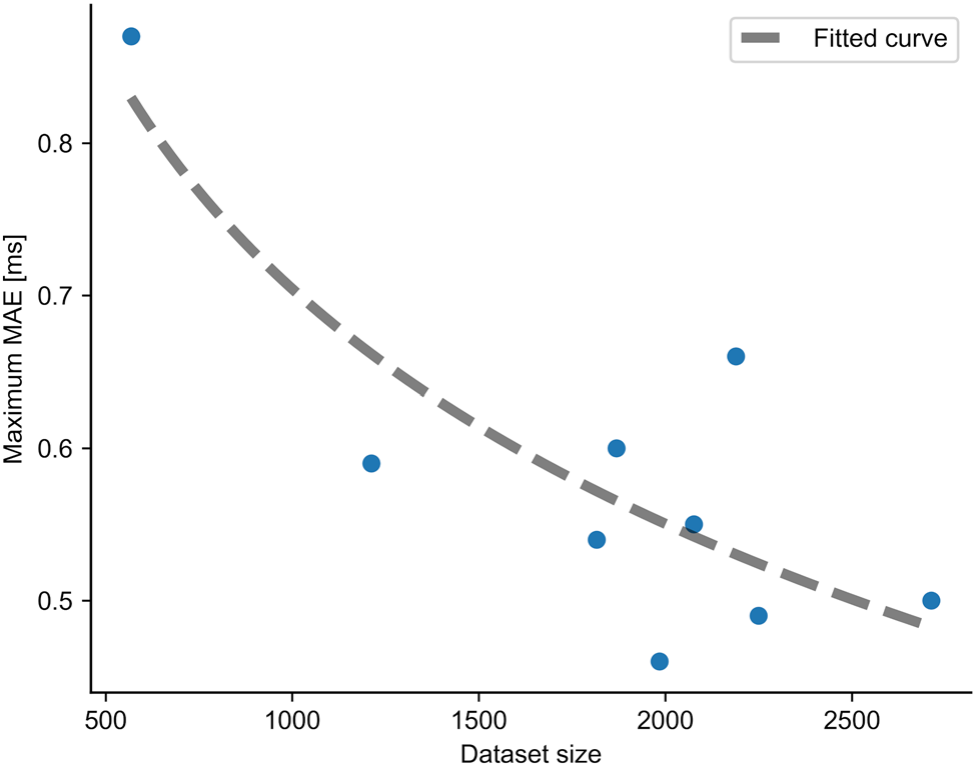
Relation between the average MAE obtained during the intra-subject cross-validation tests and the dataset size of every subject (number of MEPs). Each data point represents a different subject in the dataset.

The intra-subject variability was analyzed by computing a five-fold cross-validation using data from one subject at a time, and repeating the process for all subjects. The MAE for each data batch of each subject is reported in Table 3, together with the average and SD for all tests. Furthermore, the correlation between error and dataset size for every subject is shown in Fig. 7, together with a fitted curve.

**Table 3:** Intra-subject variability of DELMEP. A five-fold cross-validation was performed using data from one subject at a time, the process was repeated for all subjects. The table contains the mean absolute error (MAE) for every data batch of each subject, together with the average and standard deviation (SD) for every subject.

Subject Nº	Cross-validation mean absolute error (MAE) [ms]					Average MAE [ms]	SD [ms]
	1	2	3	4	5		
1	0.6	0.7	0.6	0.8	0.6	0.7	0.08
2	0.5	0.5	0.5	0.5	0.5	0.5	0.01
3	0.6	0.6	0.6	0.6	0.6	0.6	0.02
4	0.7	0.7	1.0	0.9	1.0	0.9	0.14
5	0.5	0.6	0.6	0.6	0.5	0.5	0.03
6	0.5	0.6	0.5	0.6	0.6	0.6	0.03
7	0.5	0.5	0.5	0.5	0.6	0.5	0.03
8	0.6	0.6	0.6	0.6	0.7	0.6	0.05
9	0.4	0.5	0.5	0.4	0.5	0.5	0.04

The inter-subject variability was analyzed by using the data of one of the subjects for testing and the data from the remaining eight subjects for training; this process was repeated for each subject. The MAE for each subject together with the average and SD for all tests are reported in Table 4.

**Table 4:** Inter-subject variability of DELMEP. The data from one subject was used for testing and the data from the remaining subjects for training. This process was repeated for every subject. The mean absolute error (MAE) for every subject, together with its average and standard deviation (SD) are reported.

Inter-subject mean absolute error (MAE) [ms]									Average MAE [ms]	SD [ms]
1	2	3	4	5	6	7	8	9		
0.7	0.6	0.7	0.5	0.6	0.6	0.5	0.7	0.5	0.6	0.1

**Figure 8:**
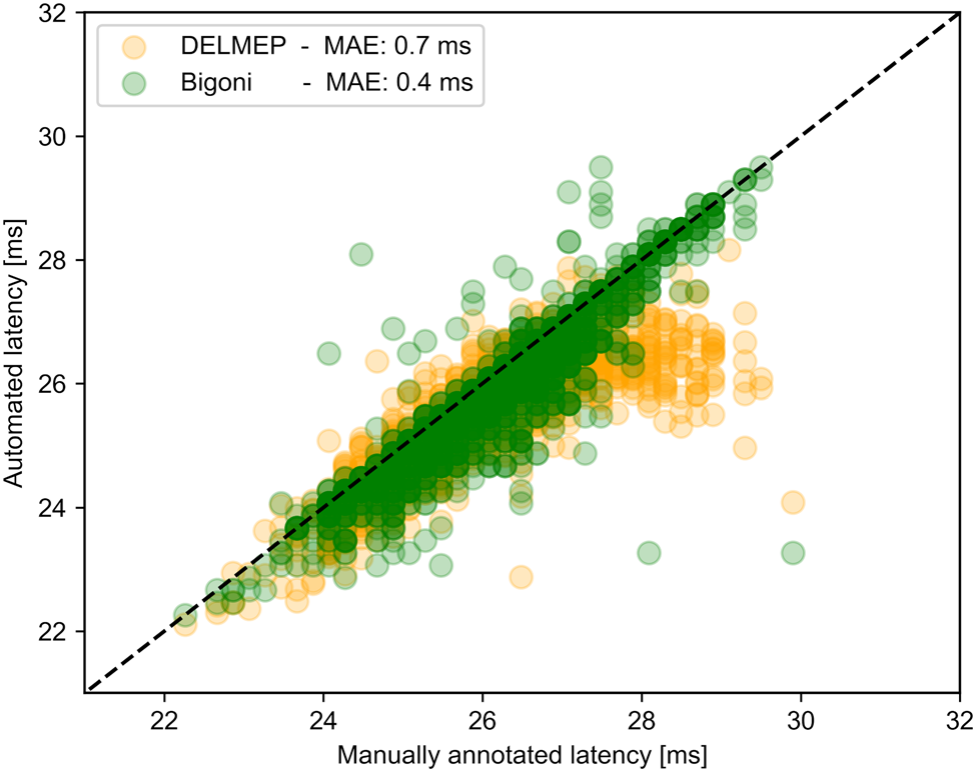
Latencies annotated with DELMEP and Bignoni’s method vs manually annotated latencies for the MEPs in Bigoni’s dataset. The manually annotated latencies are the mean value of the annotations of three independent experts.

Figure 8 shows the results for the additional validation using the dataset from Bigoni et al.^11^. The MAE from DELMEP was 0.7 ms, while that of Bigoni’s method was 0.4 ms. In the case of DELMEP, the highest errors correspond to high-latency MEPs (above 28 ms).

## Discussion

Our DELMEP algorithm performed better than traditional hard-threshold based algorithms across different MEP amplitude ranges. This improved performance is especially valuable for low-amplitude MEPs, commonly recorded at low stimulation intensities, when computing the motor threshold and in inhibitory paired-pulse paradigms^18,23,24^. For example, with the same low-amplitude MEPs (*V*_PP_ ≤ 100 µV) in the testing dataset, DELMEP, Bigoni’s method, SHTE, AHTE and Signal Hunter yielded an MAE of 0.6, 1.0, 7.3, 16.5 and 22.9 ms, respectively; with DELMEP being about one order of magnitude more accurate than these algorithms. On the other hand, with the same high-amplitude MEPs (*V*_PP_ > 100 µV) in the testing dataset, DELMEP, Bigoni’s method, SHTE, AHTE and Signal Hunter yielded an MAE of 0.5, 0.8, 1.3, 2.8 and 6.3 ms, respectively. This is possibly due to the consistent accuracy of our DELMEP algorithm regardless of the MEP amplitude. Such higher prediction errors correlated with lower MEP amplitudes can be explained by the inherently lower SNR, which has a stronger effect on methods relying on hard-threshold estimators^10^.

When tested in our larger dataset (Table 1), the automated annotation by DELMEP was on average 0.3 ms more accurate than the state-of-the-art derivative-based Bigoni’s method. Both DELMEP and Bigoni’s method retain their accuracy on low-amplitude MEPs, an important feature which is out of reach for all previously tested algorithms. When applied to Bigoni’s dataset (Fig. 8), DELMEP shows a slightly larger error than Bigoni’s method (0.7 ms and 0.4 ms, respectively). We should note that Bigoni’s method discarded 61 out of the 1561 MEPs in the dataset (approximately 4%), of which it was not able to estimate the corresponding latencies. Interestingly, the highest MAE of DELMEP corresponded to MEPs with latencies above 28 ms. This can probably be explained by the fact that the MEP dataset used to train DELMEP had latencies mostly below 28 ms (see Fig. 4), which could potentially affect the performance of the method. Nevertheless, DELMEP and Bigoni’s method show a similar accuracy for general applications and are about an order of magnitude more accurate than traditional hard-threshold algorithms.

From the user point of view, both DELMEP and Bigoni’s algorithms work by providing the MEP trace as an input and obtaining the estimated latency as an output. The machine learning nature of DELMEP makes it a more complex algorithm than Bigoni’s method. However, this does not translate into a disadvantage for the user, since the code made available with this publication is ready-to-use and no experience in machine learning is required for using it in research and/or clinical applications. Re-training DELMEP on a new training dataset requires just minor changes to the source code and a few seconds of running time in a regular desktop computer. Both algorithms require minimal human labor time and, unless modifications to the code are intended, minimal interventions and technical knowledge as well.

From a technical point of view, the main difference between DELMEP and Bigoni’s method is that the former is a machine learning algorithm, which “learns” how to annotate MEPs through a dataset of examples; while the latter is a rule-based algorithm, which finds the latency of MEPs by following a static set of steps. This makes Bigoni’s method simpler and a more explicable algorithm than DELMEP. However, an important advantage of our deep learning approach is the possibility to pre-train and apply the neural networks on application-specific datasets. For instance, separate models can be created for MEPs from the leg, forearm, and hand muscles, which naturally have distinct latencies^25,26^. Therefore, this approach may provide more accurate automated annotations for a wider set of applications. Deep learning algorithms can also be used in active-learning processes to constantly and automatically improve the accuracy of their annotations^27–29^, by periodically retraining them on data generated during their utilization. This is of special importance for applications on personalized medicine. As depicted in Fig. 7, when training and testing on data from a single subject, the latency estimation errors were noticeably reduced as the size of the available dataset was increased. Thus, the proposed DELMEP algorithm could be trained on already-available annotated MEPs of one particular subject, and thereafter used to automate the annotation of MEPs of this subject, in order to ensure the best possible accuracy. This is a feature that non-machine learning algorithms do not have, due to their static set of rules.

We should note that a deep learning-based algorithm requires a large dataset for training. However, for a research lab already performing experiments using MEPs, there might be a suitable dataset available, since just a few sessions can produce thousands of MEPs. Data from previous studies are useful even if they were recorded on different muscles and using a different setup (e.g., with a different sampling frequency or stimulation paradigm). Moreover, if more MEPs are required, there is no need for the same expert to annotate them. However, DELMEP would benefit from different experts annotating different sections of the dataset, as that would reduce the chance of the algorithm overfitting to biases that could be present in a single expert (e.g., a tendency to under- or over-estimate MEPs latencies). In this regard, we note that DELMEP was trained on MEPs annotated by a single expert. There was a 0.20 ms increase in the MAE (0.50–0.70 ms) when comparing the results from testing against the same expert used for the training, and a committee of three independent experts on a different dataset. This increase could be partially caused by having used a single expert to annotate the MEPs in the training dataset. However, this is still a state-of-the art accuracy. Moreover, using a single expert to annotate the latencies facilitates and drastically speeds up the process. On the other hand, the MAE of Bigoni’s method when tested on their own dataset versus on our dataset increased 0.40 ms (from 0.40 to 0.80 ms), indicating that a variation in accuracy of this magnitude is possible even if the algorithm is not based on machine learning. As a reference, Bigoni et al.^11^ found a difference of about 0.40 ms when comparing the estimation of two experts.

We should also emphasize that the low computational cost associated with our DELMEP algorithm allows it to be efficiently used in real-time closed-loop brain stimulation protocols^30,31^ and combined with multi-coil TMS electronic targeting for fast and automated cortical mappings^32–34^, as it requires roughly 1 ms to process an MEP in a regular desktop computer.

## Conclusions

We developed a deep learning-based algorithm to annotate MEP latencies automatically without the need for a human expert intervention. The main difference between our algorithm and previously reported solutions is that the deep learning nature of DELMEP allows it to learn and improve based on the available data, making it an ideal candidate for personalized clinical applications. The accuracy of our DELMEP algorithm was practically independent of the amplitude of the MEP, a feature only found in Bigoni’s method, as all threshold-based algorithms considered in this study failed this test. We demonstrated that DELMEP has a high accuracy on two independent datasets. The millisecond-level automated annotation in our proposed DELMEP algorithm opens a possibility for real-time assessment of MEP latencies in closed-loop brain stimulation protocols.

## Data Availability

The data will be provided upon a reasonable request (including but not limited to reproducibility and further related analysis). Requests should be sent to the corresponding author and the data confidentiality requirements should be strictly followed according to our ethical permission statement. The Python implementation of the DELMEP algorithm used in this study is available at 10.5281/zenodo.7920467 and the repository for development can be accessed at https://github.com/connect2brain/delmep.
